# Integrative analysis of transcriptomic landscape and urinary signature reveals prognostic biomarkers for clear cell renal cell carcinoma

**DOI:** 10.3389/fonc.2023.1102623

**Published:** 2023-03-24

**Authors:** Wei Zhang, Wenqiang Liu, Yiren Yang, Chengwu Xiao, Yutian Xiao, Xiaojie Tan, Qingyang Pang, Han Wu, Meimian Hua, Xiaolei Shi

**Affiliations:** ^1^ Department of Urology, Changhai Hospital, Naval Medical University, Shanghai, China; ^2^ Department of Epidemiology, Naval Medical University, Shanghai, China

**Keywords:** clear cell renal cell carcinoma, venous tumor thrombus, prognosis, urine, biomarker

## Abstract

**Background:**

Clear cell renal cell carcinoma (ccRCC) patients with venous tumor thrombus (VTT) have poor prognosis. We aimed to reveal features of ccRCC with VTT and develop a urine-based prognostic classifier to predict ccRCC prognosis through integrative analyses of transcriptomic landscape and urinary signature.

**Methods:**

RNA sequencing was performed in five patients with ccRCC thrombus-tumor-normal tissue triples, while mass spectrometry was performed for urine samples from 12 ccRCC and 11 healthy controls. A urine-based classifier consisting of three proteins was developed to predict patients’ survival and validated in an independent cohort.

**Results:**

Transcriptomic analysis identified 856 invasion-associated differentially expressed genes (DEGs). Furthermore, proteomic analysis showed 133 differentially expressed proteins (DEPs). Integration of transcriptomic landscape and urinary signature reveals 6 urinary detectable proteins (VSIG4, C3, GAL3ST1, TGFBI, AKR1C3, P4HB) displaying abundance changes consistent with corresponding genes in transcriptomic profiling. According to TCGA database, VSIG4, TGFBI, and P4HB were significantly overexpressed in patients with shorter survival and might be independent prognostic factors for ccRCC (all p<0.05). A prognostic classifier consisting of the three DEPs highly associated with survival performed satisfactorily in predicting overall survival (HR=2.0, p<0.01) and disease-free survival (HR=1.6, p<0.001) of ccRCC patients. The ELISA analysis of urine samples from an independent cohort confirmed the satisfied predictive power of the classifier for pathological grade (AUC=0.795, p<0.001) and stage (AUC=0.894, p<0.001).

**Conclusion:**

Based on integrative analyses of transcriptomic landscape and urinary signature, the urine-based prognostic classifier consisting of VSIG4, TGFBI, and P4HB has satisfied predictive power of ccRCC prognosis and may facilitate ccRCC molecular subtyping and treatment.

## Introduction

Renal cell carcinoma (RCC) is a frequently diagnosed cancer originating from the renal epithelium, with an estimated 431,280 new incidences globally in 2020 (1). RCC comprises a heterogeneous group of malignant tumors, of which the most common (~70%) and aggressive histological subtype is clear cell RCC (ccRCC) (2). ccRCC is prone to metastasis, as about 30% of the patients have metastasis at the first visit, and one-third of the remaining patients have recurrence and metastasis after surgery (3, 4). In addition, 4%-15% of the patients have their primary tumor invading the venous system to form venous tumor thrombus (VTT). The ccRCC patients with VTT exhibit poor prognosis if left untreated, with a 5-year disease-specific survival rate of 10% (2, 5). The current first-line regimen for metastatic and locally advanced ccRCC is immune checkpoint inhibitor combined with tyrosine kinase inhibitor (6). Although it has greatly improved the survival of ccRCC patients, the acquired resistance after receiving treatment or even original drug resistance are still challenges (7–9). Timely identification of these cases would improve the overall survival (OS) of ccRCC patients.

At present, the risk stratification and prognosis prediction models in current clinical practice are mainly pathological characteristics including WHO/ISUP grades and TNM stages (6). However, patients with similar clinical and pathological features may have different prognosis in that ccRCC exhibited extensive functional and genomic intratumoral heterogeneity (10, 11). Therefore, it is urgent to discover those molecular markers related to prognosis, so as to develop a prognostic classifier to facilitate ccRCC molecular subtyping and treatment. As an important method of liquid biopsy, urine is the ideal biological matrix for discovery of cancer biomarkers, in particular for kidney-related diagnostics (12). In addition, its non-invasive and cost-effective natures make it suitable for providing a personalized snapshot of disease during active surveillance or postoperative follow-up (13).

In the study, we first reveal features of ccRCC with VTT through integrative analyses of transcriptomic landscape and urinary signature. Second, a urine-based prognostic classifier consisting of the prognosis-related proteins was developed to predict ccRCC prognosis. Finally, the predictive efficiency of this prognostic classifier was further validated by ELISA analysis of urine samples from an independent cohort to facilitate ccRCC molecular subtyping.

## Materials and methods

### Patient selection and sample collection

**Figure 1:**
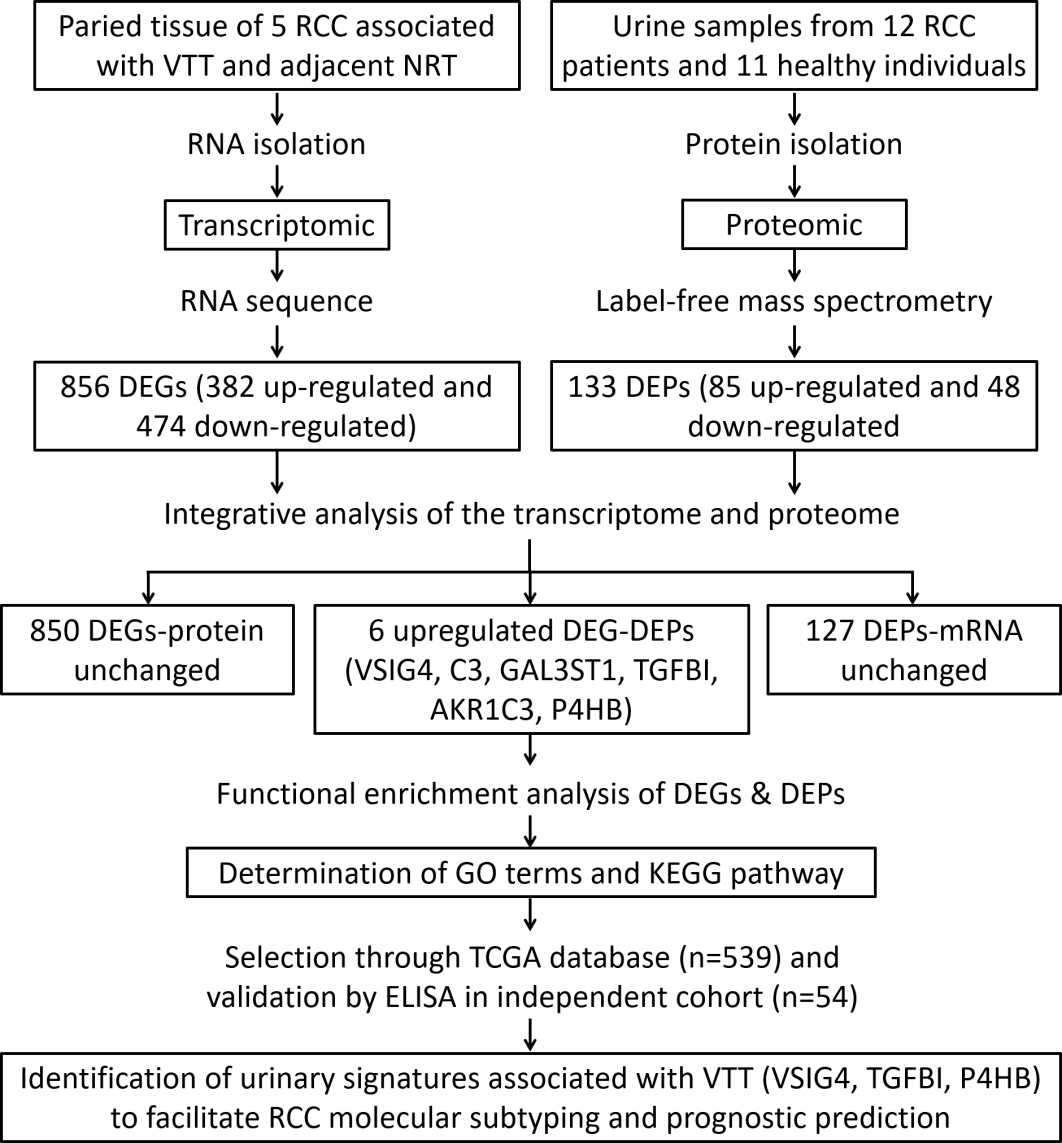
Flowchart of RNA sequencing in ccRCC patients with thrombus-tumor-normal tissue triples and mass spectrometry in urine samples from ccRCC patients and healthy controls to develop a urine-based prognostic classifier for predicting ccRCC prognosis.

For RNA sequencing, patients were included if they had histologically confirmed ccRCC with VTT. The ccRCC thrombus-tumor-normal tissue triples of 5 cases were obtained following nephrectomy and tumor thrombus resection (**Supplementary Table 1**). For mass spectrometry, 12 patients with histological-type ccRCC undergoing nephrectomy and 11 healthy donor volunteers from the same period were included (**Supplementary Table 2**). Their samples of the second urine in the morning were collected before surgery in sterile tubes containing 1 mM of phenylmethanesulfonyl fluoride (Sigma, St. Louis, MO) to inhibit proteases. In addition, 54 urine samples from an independent cohort of consecutive ccRCC patients were also collected for ELISA analysis (**Supplementary Table 3**). **Figure 1** shows a workflow summary of the transcriptomic and proteomic research that revealed characteristics of ccRCC with VTT and developed a urine-based prognostic classifier to predict ccRCC prognosis. The study was approved by the ethics committee of Changhai Hospital, Naval Medical University, and written informed consent was obtained from all participants prior to study enrollment.

### RNA sequencing

Total RNA of thrombus, tumor and normal tissue from ccRCC patients was extracted using the mirVana miRNA Isolation Kit (Ambion, TX, USA) following the manufacturer’s instructions. RNA purity was checked using a NanoPhotometer spectrophotometer (IMPLEN, CA, USA). The TruSeq Stranded mRNA LTSample Prep Kit (Illumina, CA, USA) was used to build the libraries. Then these libraries were sequenced on the Illumina sequencing platform (HiSeqTM 2500 or Illumina HiSeq X Ten) and 150 bp paired-end reads were generated.

### Mass spectrometry

The urine samples were centrifugated to collect the supernatant, and then the protein extract in urine supernatant was digested into peptides with trypsin. The peptides were subjected to capillary source followed by the timsTOF Pro (Bruker Daltonics) mass spectrometry. The electrospray voltage applied was 1.60 kV. Precursors and fragments were analyzed at the TOF detector, with a MS/MS scan range from 100-1700 m/z. The timsTOF Pro was operated in parallel accumulation serial fragmentation (PASEF) mode. Precursors with charge states 0 to 5 were selected for fragmentation, and 10 PASEF-MS/MS scans were acquired per cycle. The dynamic exclusion was set to 30s.

### Analyses of differentially expressed genes/proteins

The analyses of differentially expressed genes (DEGs) and differentially expressed proteins (DEPs) were performed using the “limma” package of R statistical software.

DEGs were divided among three groups: RCC vs. normal renal tissue (NRT), VTT vs. NRT, VTT vs. RCC. The DEGs which co-expressed in RCC vs. NRT and VTT vs. NRT and those in VTT vs. RCC were defined as thrombus invasion-associated genes. Furthermore, DEPs were selected based on their different levels between urinary samples of ccRCC patients and healthy controls. DEGs/DEPs were defined by |log2 FC|>2 and P<0.05. For the public single-cell RNA sequencing data, the transcriptional profiles from all ccRCC patients and samples were visualized *via* uniform manifold approximation and projection. Then, the normalized expressions of DEGs were presented in all single-cell clusters and compared among tissues of ccRCC tumor, adjacent normal kidney, and lymph node. Gene Ontology (GO) functional annotation and Kyoto Encyclopedia of Genes and Genomes (KEGG) pathway enrichment were performed using the “clusterProfiler” package of R statistical software.

### Screening of prognostic proteins for survival

Using the survival package, the univariate Cox regression analysis was carried out to targeted proteins linked to OS. (version 3.3.1; https://github.com/therneau/survival). The optimal prognostic protein set for OS was further screened on the basis of SVM-RFE method using the e1071 (version 1.7.1; https://cran.r-project.org/web/packages/e1071) and caret packages (version 6.0.76; https://cran.r-project.org/web/packages/caret). The SVM classifier was then built to predict OS according to the expression levels of optimal prognostic protein set. Additionally, the results of the SVM classification analysis were validated using data from The Cancer Genome Atlas-Kidney Renal Clear Cell Carcinoma (TCGA-KIRC) dataset.

### Development and validation of prognostic classifier for survival

The multivariate Cox regression analysis was performed to extract independent prognostic genes for OS using survival package (version 3.3.1; https://github.com/therneau/survival). Afterwards, a risk score model of prognostic makers was established according to following formula: risk score = ∑βDEPs × ExpDEPs. The βDEPs represented the estimated contribution coefficient of independent prognostic proteins in multivariate Cox regression analysis and ExpDEPs denoted the level of independent prognostic genes. Then, all patients were divided into high- or low-risk groups with the median of risk scores as the cutoff.

### Statistical analysis

All data processing and statistical tests were performed using R 4.1.2 and further visualized using GraphPad Prism 6. The continuous parametric variables were displayed as mean ± standard deviation and compared using Student’s t-Test. The hazard ratios (ORs) and corresponding 95% confidence intervals (CIs) of the selected predictors of survival were also presented. The difference in survival between two groups was shown with Kaplan-Meier curves, and the receiver operating characteristic curve (ROC) for pathological grades and stages were drawn to obtain the area under the curve (AUC) values. Statistically significant P value was set at 0.05 with two sides.

## Results

### Transcriptomic landscape and urinary signature of ccRCC patients with VTT

**Figure 2:**
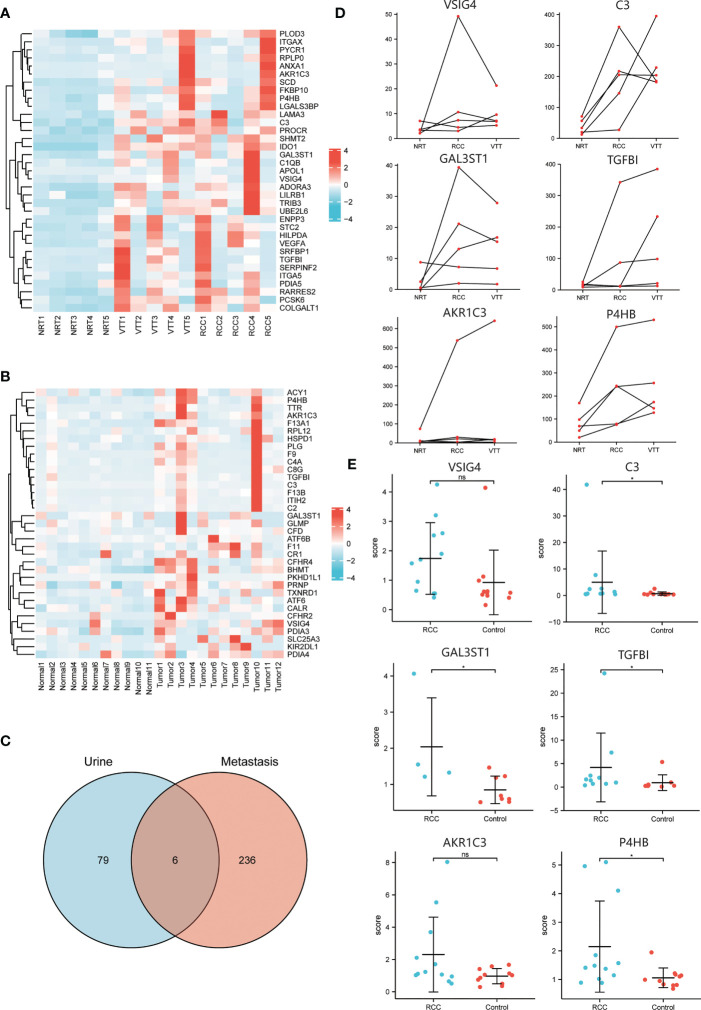
The integrative analysis data of transcriptomic landscape and urinary signature in ccRCC patients. **(A)** Heatmap of DEGs in transcriptome analysis of normal, tumor and thrombus tissue showing the top up-regulated thrombus invasion-associated genes. **(B)** Heatmap of DEPs in proteome analysis of urine samples from ccRCC patients and healthy controls showing the top up-regulated proteins. **(C)** Venn diagram to illustrate the six urinary detectable proteins (VSIG4, C3, GAL3ST1, TGFBI, AKR1C3, P4HB) displaying abundance changes consistent with corresponding genes in transcriptomic profiling. **(D)** Regulative expression trends of DEGs among normal, tumor and thrombus tissue indicating expressions of TGFBI, AKR1C3, P4HB increase consecutively from NRT to RCC and then to VTT. **(E)** Different expressions of DEPs in urine between ccRCC patients and healthy controls indicating expressions of VSIG4, C3, GAL3ST1, TGFBI, AKR1C3, P4HB in ccRCC patients are over 1.5-time higher than those in healthy controls. *p < 0.05, ns, no significance.

The transcriptomic analysis of 5 matched RCC, VTT and NRT tissues found 1131, 1258, and 63 transcripts differentially expressed in RCC vs. NRT, VTT vs. NRT, and VTT vs. RCC groups, respectively. Among them, 856 DEGs were obtained as thrombus invasion-associated genes, of which there were 382 up-regulated and 474 down-regulated genes (**Figure 2A**). In addition, mass spectrometry analysis of urinary samples between 12 ccRCC patients and 11 healthy subjects showed 133 DEPs, with 85 up-regulated and 48 down-regulated proteins (**Figure 2B**).

The integrative analysis of transcriptomic landscape and urinary signature reveals six urinary detectable proteins (VSIG4, C3, GAL3ST1, TGFBI, AKR1C3, P4HB) displaying upregulated abundance changes consistent with corresponding genes in transcriptomic profiling (**Figure 2C**). Among them, expressions of TGFBI, AKR1C3, and P4HB increased consecutively from NRT to RCC and then to VTT, indicating that they had a consistent promoting effect in the processes of tumorigenesis and thrombus invasion (**Figure 2D**). The expressions of the targeted proteins in urine samples of ccRCC patients were over 1.5-time higher than those of healthy controls. However, only the expressions of C3, GAL3ST1, TGFBI, and P4HB achieved statistically significant difference between two groups (**Figure 2E**).

### The upregulated DEPs indicate poor survival in ccRCC patients

**Figure 3:**
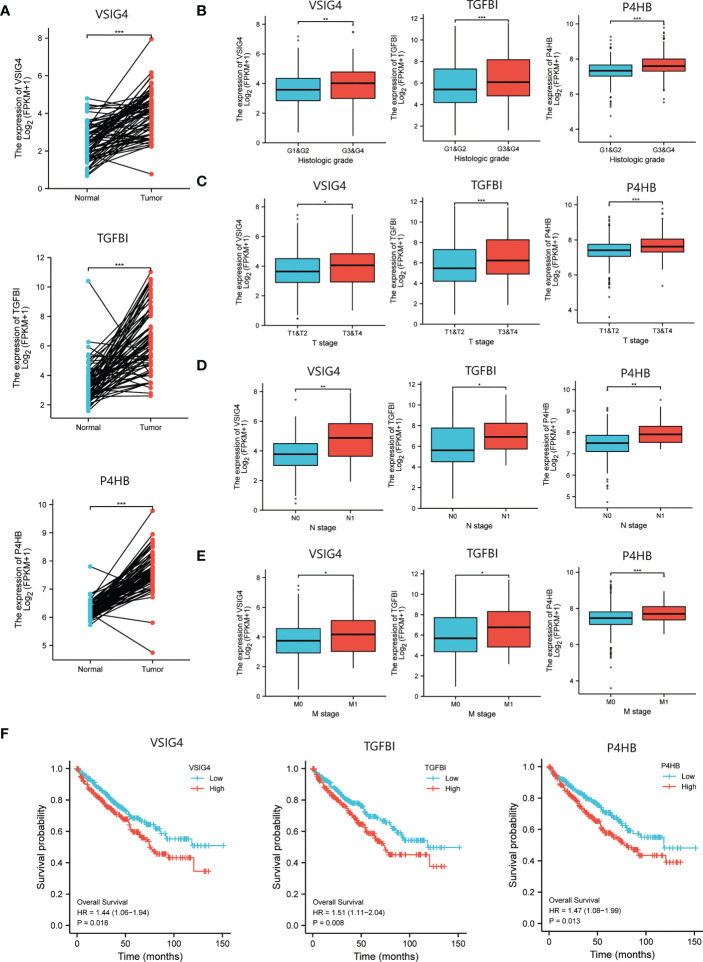
The selection of prognosis-related molecules based on TCGA database. **(A)** Different expressions of the prognosis-related genes between ccRCC tumor and normal renal tissue indicating higher mRNA levels of VSIG4, TGFBI, P4HB in tumor compared to matched normal tissue. **(B–E)** Different expressions of the prognosis-related genes between ccRCC patients with different pathological characteristics including WHO/ISUP grades and TNM stages indicating increased mRNA levels of VSIG4, TGFBI, P4HB are associated with higher pathological grades and later pathological stages. **(F)** The Kaplan-Meier curves of OS for ccRCC patients with different expressions of the prognosis-related genes showing VSIG4, TGFBI, P4HB are overexpressed in patients with shorter survival. *p < 0.05, **p < 0.01, ***p < 0.001.

We obtained the transcriptional and follow-up data from TCGA and evaluated the correlation between expressions of targeted proteins and prognosis of ccRCC patients. First, the significant higher mRNA levels of all the six proteins in tumor compared to matched normal renal tissue were verified (**Figure 3A**; **Supplementary Figure 1A**). Second, in the TCGA cohort of ccRCC patients, increased mRNA levels of VSIG4, TGFBI, P4HB were associated with higher pathological grades (all p<0.01) and later pathological stages (all p<0.05) (**Figures 3B-E**). While mRNA levels of C3, AKR1C3, GAL3ST1 were not completely correlated with tumor pathological grades and stages (**Supplementary Figures 1B-E**). Third, significant expression differences of VSIG4, TGFBI, and P4HB could be seen between patients with different OS events (366 alive vs. 173 dead). They were significantly overexpressed in patients with shorter survival and might be independent prognostic factors for ccRCC patients (all p<0.05) (**Figure 3F**). However, the expression differences of C3, AKR1C3, and GAL3ST1 were not seen in ccRCC patients with different prognosis (**Supplementary Figure 1F**).

### A urine-based prognostic classifier to predict ccRCC prognosis

**Figure 4:**
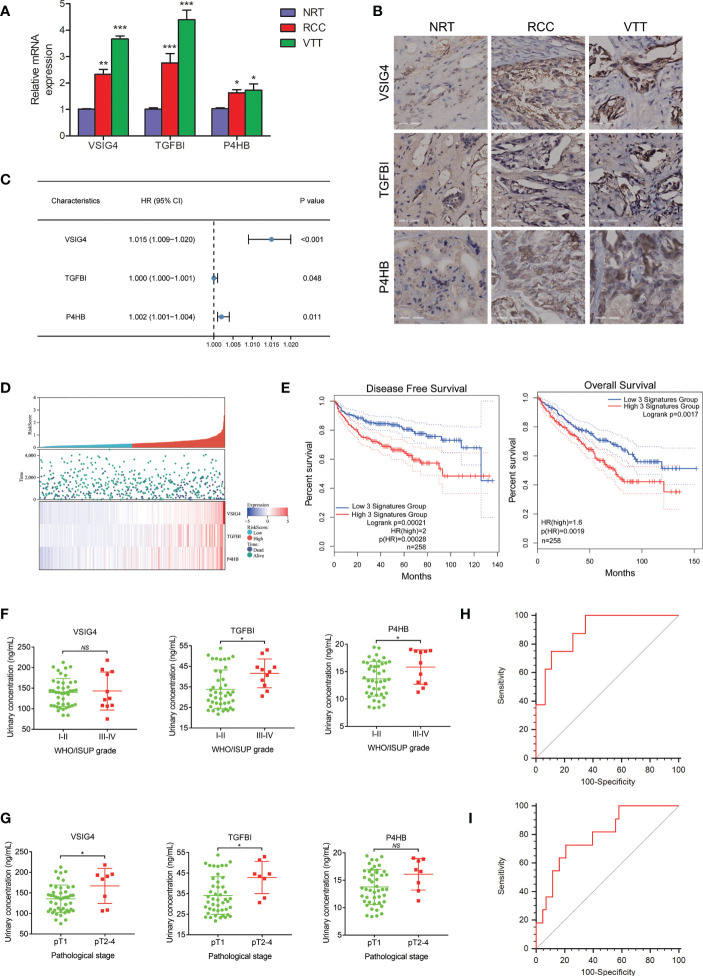
The development and validation of a urine-based prognostic classifier for survival. **(A)** qRT-PCR analysis of the selected prognosis-related molecules in ccRCC thrombus-tumor-normal tissue triples showing mRNA levels of VSIG4, TGFBI, P4HB are the highest in VTT, followed by those in RCC and NRT. **(B)** IHC analysis of the selected prognosis-related molecules in ccRCC thrombus-tumor-normal tissue triples showing protein expressions of VSIG4, TGFBI, P4HB increase consecutively from NRT to RCC and then to VTT. **(C)** Forest plot of hazard ratios for the genes in prognostic classifier showing expressions of VSIG4, TGFBI, P4HB are highly associated with survival. **(D)** Distributions of risk score and expression profile of the genes in prognostic classifier in patients with different survival time and status. **(E)** The Kaplan-Meier curves of OS and DFS for ccRCC patients in high-risk and low-risk groups by prognostic classifier in TCGA database showing patients in high-risk group had shorter OS and DFS time. **(F, G)** Different urinary expressions of the proteins in prognostic classifier between ccRCC patients with different pathological grades and stages indicating urinary TGFBI and P4HB are overexpressed in patients with higher grade tumors while urinary VSIG4 and TGFBI are overexpressed in patients with later pathological stages. **(H, I)** The ROCs for the prognostic classifier predicting pathological grade and stage of ccRCC patients by ELISA showing AUC value of 0.795 for pathological grade and AUC value of 0.894 for pathological stage. *p < 0.05, **p < 0.01, ***p < 0.001, NS, no significance.

The qRT-PCR and immunohistochemistry (IHC) experiments were respectively conducted to evaluate the mRNA and protein expression levels of VSIG4, TGFBI, and P4HB in ccRCC thrombus-tumor-normal tissue triples. The qRT-PCR analysis showed that mRNA levels of these three molecules were the highest in VTT, and then their levels in RCC were significantly higher than those in NRT (**Figure 4A**). The IHC assay further confirmed that protein expressions of VSIG4, TGFBI, and P4HB increased consecutively from normal kidney to renal tumor and then to tumor thrombus (**Figure 4B**).

The three proteins highly associated with survival (VSIG4, TGFBI, and P4HB) were used to establish a prognostic classifier (**Figure 4C**). We calculated the risk score of survival in each case from TCGA database according to expression levels of these three proteins, and then divided patients into high- or low-risk groups (**Figure 4D**). It demonstrated that ccRCC patients in high-risk group had shorter OS time (HR=2.0, p<0.01) and disease-free survival (DFS) time (HR=1.6, p<0.001) (**Figure 4E**).

The ELISA analysis was conducted in 54 urine samples from an independent cohort of ccRCC patients. As for the tumor pathological characteristics, the WHO/ISUP grade was I in two cases, II in 41 cases, III in nine cases, and IV in two cases. Urinary detectable TGFBI and P4HB, but not VSIG4, were demonstrated to be higher expressed in patients with III-IV grade tumor than those with I-II grade tumor (**Figure 4F**). The T stage was T1a in 36 cases, T1b in nine cases, T2 in three cases, and T3-4 in six cases. Urinary detectable VSIG4 and TGFBI, but not P4HB, were demonstrated to be higher expressed in patients with pathological T2-4 stage than those with pathological T1 stage (**Figure 4G**). Finally, it confirmed the satisfactory predictive power of the prognostic classifier for pathological grade (AUC=0.795, p<0.001) (**Figure 4H**) and stage (AUC=0.894, p<0.001) (**Figure 4I**) in ccRCC patients.

### Effects of DEPs on tumor microenvironment and thrombus invasion

**Figure 5:**
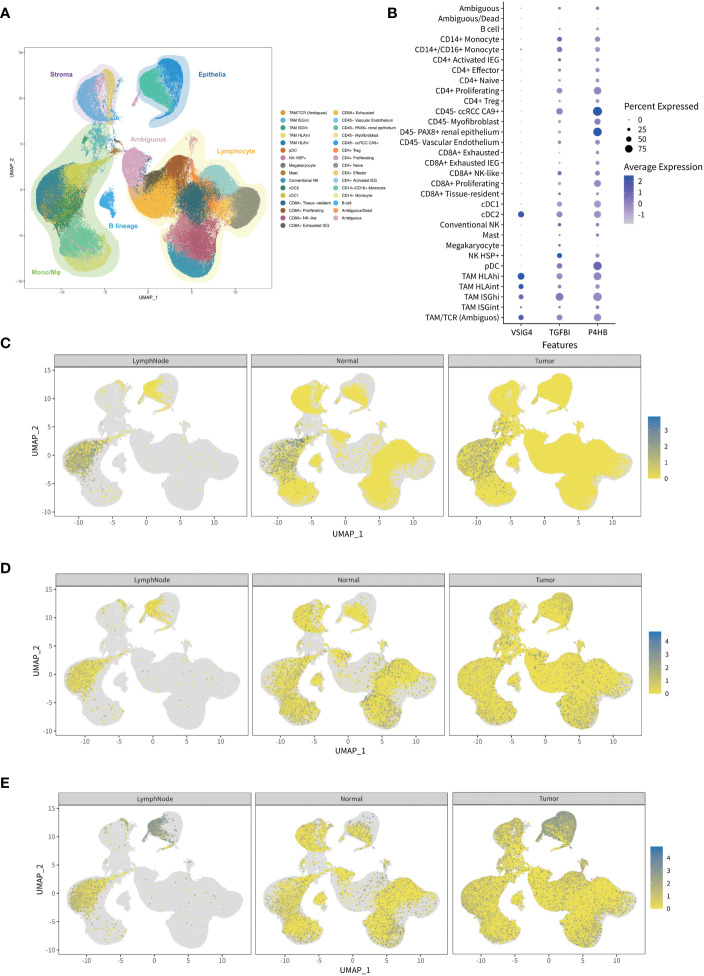
The expression analysis of the genes in prognostic classifier through single-cell RNA sequencing public database. **(A)** Visualized map of transcriptional profiles from all ccRCC patients and samples showing 31 single-cell clusters including lymphoid, myeloid, epithelial cells, and cancer cells. **(B)** Normalized expressions of the genes in prognostic classifier among 31 single-cell clusters indicating VSIG4 is uniquely expressed in tumor-associated macrophages while TGFBI and P4HB are broadly expressed. **(C–E)** Comparison of gene expressions among ccRCC tumor, adjacent normal kidney, and lymph node indicating macrophage-expressed VSIG4 is higher in lymph node than tumor while epithelium-expressed TGFBI and P4HB are higher in tumor than normal kidney.

To determine the key roles of selected proteins in processes of tumorigenesis and thrombus invasion, we analyze the single-cell RNA-sequencing data obtained from research by Krishna et al (14). Louvain clustering revealed 31 clusters across tissues spanning lymphoid, myeloid, epithelial cells, and cancer cells based on the single-cell RNA-sequencing of 167,283 cells from multiple tumor regions, lymph node, normal kidney of ccRCC patients (**Figure 5A**). VSIG4 was indicated to be a characteristic marker for tumor-associated macrophage populations, while TGFBI and P4HB were showed to be broadly expressed in ccRCC tumor and its immune microenvironment. Furthermore, the average expression level of P4HB in ccRCC tumor and renal epithelium was the highest among 31 single-cell clusters (**Figure 5B**). After dividing single-cell transcriptomes into ccRCC tumor, adjacent normal kidney, and lymph node subgroup according to the different sources of each cell. As we can see, the macrophage-expressed VSIG4 in lymph node was higher than that in ccRCC tumor and adjacent normal kidney (**Figure 5C**), whereas the epithelium-expressed TGFBI and P4HB in ccRCC tumor were higher than those in adjacent normal kidney (**Figures 5D, E**). In addition, the GO and KEGG enrichment analyses disclosed that those selected proteins were predominantly related to the central carbon metabolism, ferroptosis, ECM-receptor interaction, and platinum drug resistance (**Supplementary Figure 2**).

## Discussion

The omics-based analytical approaches are becoming available to enhance the understanding of the tumor pathophysiology (15, 16). Transcriptomic technique focuses on coding and noncoding sequences to identify differentially expressed genes. While proteomic approach makes it an ideal strategy to study the molecular mechanism of RCC. However, given the complexity and variability of the pathophysiological processes involved in RCC, independent analysis from each omics level may miss crosstalk between different molecular entities and biological relevant information (17, 18). In this context, integrated analysis has emerged as a novel approach that facilitate interpretation of multidimensional data and insights into extensive functional and genomic intratumoral heterogeneity in RCC. The ccRCC patients with/without VTT show distinct molecular characteristics in that tumors from ccRCC patients with VTT showed a higher mutational burden and genomic instability (19). Furthermore, macrophages, malignant cells, endothelial cells and myofibroblasts in VTT exhibited enhanced remodeling of the extracellular matrix pathways compared to matched primary cancer cells, providing evidence of phenotypic heterogeneity between primary tumors and tumor thrombus (20). To our knowledge, there have been few studies depicting RCC infiltration into the renal vein by tumor thrombus-related multi-omics analysis (21).

As the number of prognostic biomarkers for ccRCC has been increasing regularly over the last decade, Petitprez et al. (22) performed a review of the relevant studies and found that the predictive methods have evolved from single markers to multiple-marker models. Interestingly, the main genes involved in ccRCC carcinogenesis such as VHL, PBRM1, BAP1, and SETD2, were not the most relevant for predicting survival. Our results suggest that in addition to body biofluid samples including plasma and urine, thrombosis may also contain biomarker information related to the prognosis of ccRCC patients, which can provide new ideas for the discovery of biomarkers. In addition, the constructed prognostic classifier in our study can be detected in urinary specimens. The urine carries a variety of set of soluble proteins and peptides that are primarily derived from kidney, bladder and prostate (23). Chinello et al. (24) conducted integrative proteomic analyses of the urine and blood in ccRCC patients and found that urine carried specific “biofluid functional signature”, which provided a landscape of RCC dynamic system of processes in venous infiltration. One major advantage of urinary biomarkers is that the detection of these markers is noninvasive, convenient, high-volume, and easy to evaluate. Thus, this liquid biopsy method can be scheduled frequently to provide a personalized snapshot of disease to actively monitor disease progression. Such narrow control also allows a rapid switch in the case for therapy by any changes (13). In our study, satisfactory predictive power of the urine-based prognostic classifier for pathological grade and stage of ccRCC was finally verified through ELISA analysis of 54 urine samples from an independent cohort.

The review of 341 reported prognostic biomarkers in ccRCC found that 20% of these biomarkers were involved in four biological pathways: hypoxia, angiogenesis, cell cycle, and immune response (22). In terms of the biological activities of the dysregulated thrombus invasion-associated genes in our study, several *in vitro* experiments showed that TGFBI promoted adhesion, migration, and invasion in ccRCC cells (25, 26). Recent study further showed that TGFBI were ubiquitinated and downregulated by VHL restoration and upregulated in human ccRCC (27). M2-related factor frequencies were regarded as robust biomarkers for predicting the renal clear cell carcinoma patient clinical phenotype and immune microenvironment. Wang et al. explored M2 macrophage-related factors of ccRCC and found that VSIG4, as a co-expressed gene of M2 macrophages, was correlated with infiltration of M2 macrophages and predicted outcomes of ccRCC (28). As an autophagy-related gene, P4HB was proposed to be one potential novel ccRCC diagnostic and prognostic biomarker at both mRNA and protein levels (29, 30). Furthermore, P4HB could be used to construct prognostic models with other autophagy-related genes or clinicopathological parameters (31). However, the role of P4HB in occurrence and invasion processes of ccRCC has not been reported. Further studies on biological processes associated with these molecules would expand applications of our prognostic classifier including prediction of patient response to targeted therapy or immunotherapy and discovery of novel therapeutic targets.

We do acknowledge some limitations of the study. First, the independent cohort applied to validate the performance of our prognostic classifier lacked survival information of patients. Second, the study was conducted in a single-center with limited sample size, further multicenter studies for validation are needed. Last, the biological functions of these proteins in tumorigenesis and invasion processes of ccRCC need to be revealed in the future.

## Conclusion

Based on integrative analyses of transcriptomic landscape and urinary signature, the urine-based prognostic classifier consisting of VSIG4, TGFBI, and P4HB has satisfied predictive power of survival time, pathological grade and stage in ccRCC patients, which facilitate ccRCC molecular subtyping and treatment.

## Data availability statement

The data presented in the study are deposited in the Genome Sequence Archive (Genomics, Proteomics & Bioinformatics 2021) in National Genomics Data Center (Nucleic Acids Res 2022), China National Center for Bioinformation / Beijing Institute of Genomics, Chinese Academy of Sciences repository, accession number PRJCA012759.

## Ethics statement

Written informed consent was obtained from the individual(s) for the publication of any potentially identifiable images or data included in this article.

## Author contributions

Study concept and design: WZ, MH, XS. Acquisition of data: WZ, WL, YY, CX, XS. Analysis and interpretation of data: WZ, WL,YY, YX, QP, XS Drafting of the manuscript: WZ, WL Critical revision of the manuscript for important intellectual content: MH, XS. Statistical analysis: YY, XT, HW. Administrative, technical, or material support: XT, HW. Supervision: MH, XS. All authors contributed to the article and approved the submitted version.

## Supplementary material

Supplementary Figure 1 The exclusion of prognosis-unrelated molecules based on TCGA database. **(A)** Different expressions of the prognosis-unrelated genes between ccRCC tumor and normal renal tissue indicating higher mRNA levels of C3, AKR1C3, GAL3ST1 in tumor compared to matched normal tissue. **(B-E)** Different expressions of the prognosis-unrelated genes between ccRCC patients with different pathological characteristics including WHO/ISUP grades and TNM stages indicating mRNA levels of C3, AKR1C3, GAL3ST1 are not completely correlated with tumor pathological grades and stages. **(F)** The Kaplan-Meier curves of OS for ccRCC patients with different expressions of the prognosis-unrelated genes showing no difference exists in expressions of C3, AKR1C3, GAL3ST1 between ccRCC patients with different survivals. *p < 0.05, **p < 0.01, ***p < 0.001.

Supplementary Figure 2 GO and KEGG analyses of the transcriptome. **(A)** The GO functional annotation of the genes in prognostic classifier. **(B)** KEGG pathway annotation of the genes in prognostic classifier.
