# Supplementary figures and images for: Integrative analysis of transcriptomic landscape and urinary signature reveals prognostic biomarkers for clear cell renal cell carcinoma
Source: Front Oncol. 2023 Mar 24;13:1102623. doi: 10.3389/fonc.2023.1102623 (PMC10079990; doi:10.3389/fonc.2023.1102623)

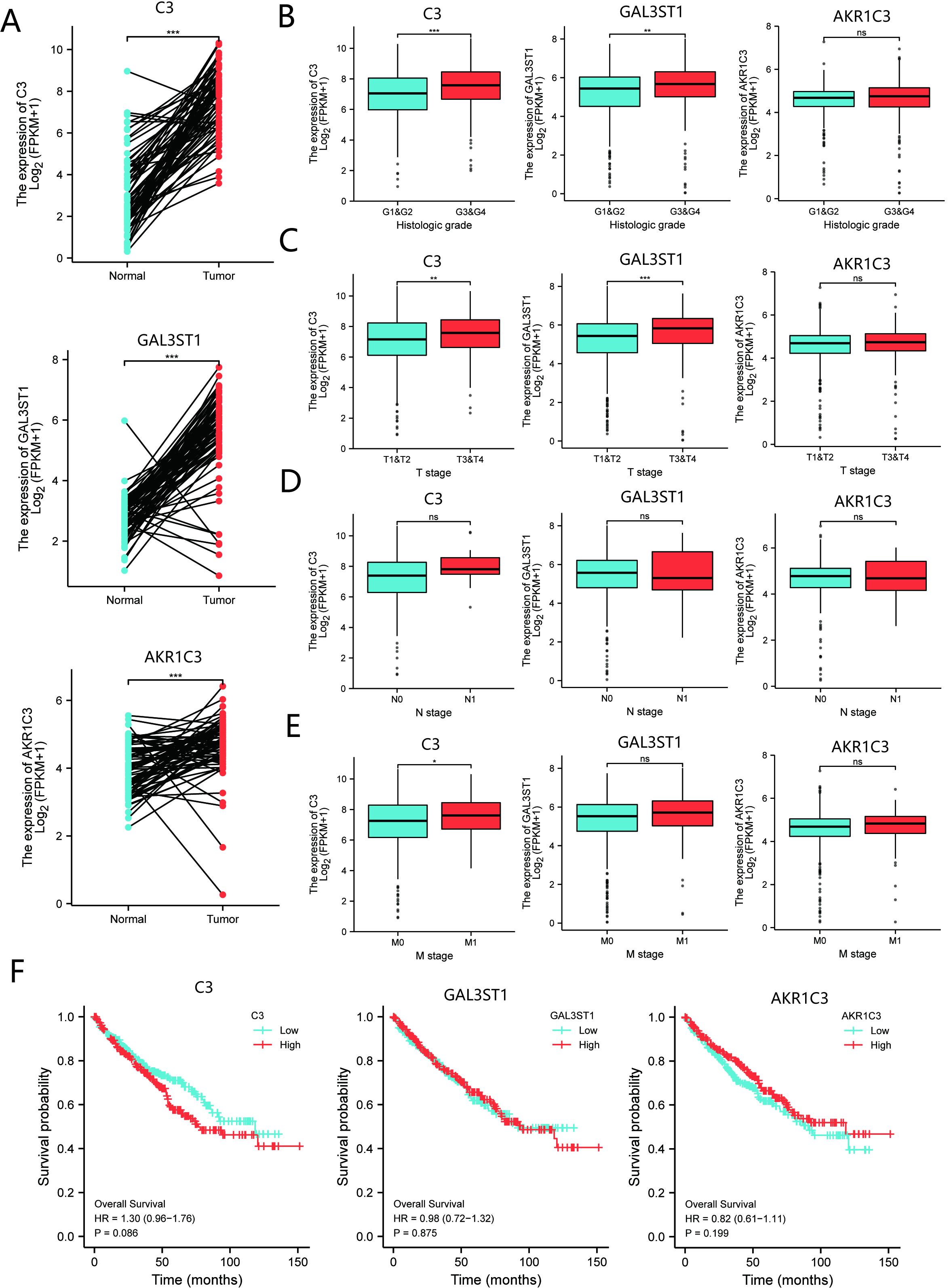

Supplement: Supplementary Figure 1 — The exclusion of prognosis-unrelated molecules based on TCGA database. (A) Different expressions of the prognosis-unrelated genes between ccRCC tumor and normal renal tissue indicating higher mRNA levels of C3, AKR1C3, GAL3ST1 in tumor compared to matched normal tissue. (B-E) Different expressions of the prognosis-unrelated genes between ccRCC patients with different pathological characteristics including WHO/ISUP grades and TNM stages indicating mRNA levels of C3, AKR1C3, GAL3ST1 are not completely correlated with tumor pathological grades and stages. (F) The Kaplan-Meier curves of OS for ccRCC patients with different expressions of the prognosis-unrelated genes showing no difference exists in expressions of C3, AKR1C3, GAL3ST1 between ccRCC patients with different survivals. *p < 0.05, **p < 0.01, ***p < 0.001. [file Image_1.tif]

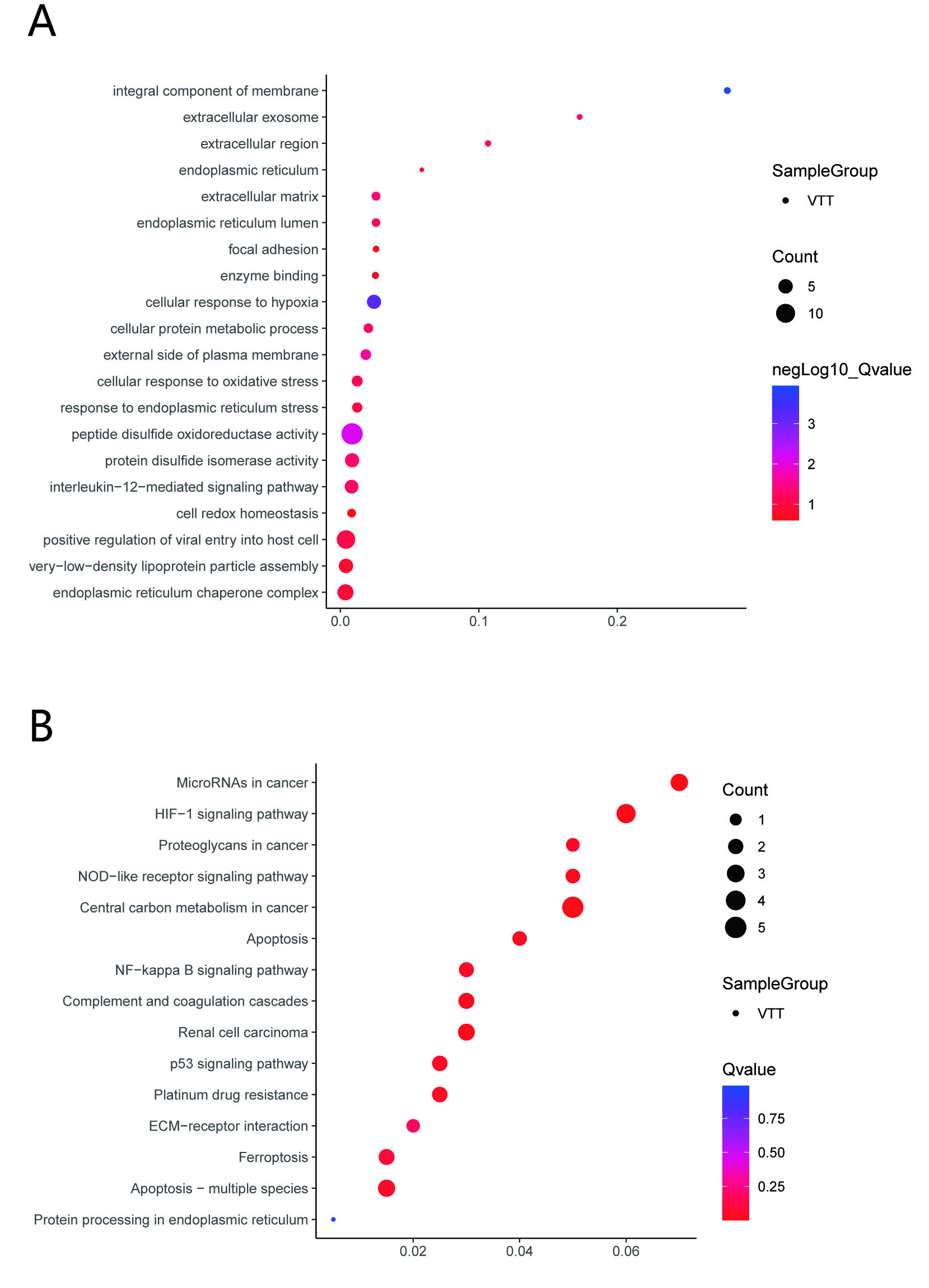

Supplement: Supplementary Figure 2 — GO and KEGG analyses of the transcriptome. (A) The GO functional annotation of the genes in prognostic classifier. (B) KEGG pathway annotation of the genes in prognostic classifier. [file Image_2.tif]
