# Supplementary material for: Integrative analysis of transcriptomic landscape and urinary signature reveals prognostic biomarkers for clear cell renal cell carcinoma
Source: Front Oncol. 2023 Mar 24;13:1102623. doi: 10.3389/fonc.2023.1102623 (PMC10079990; doi:10.3389/fonc.2023.1102623)
Supplement: Supplementary file 4 [file Table_2.docx]

Supplementary Table 1. Baseline characteristics of ccRCC patients for tissue RNA sequencing

| No. | Gender | Age (years) | BMI  (kg/m^2^) | Tumor laterality | WHO/ISUP grade | Renal mass diameter (cm) | Thrombus location |
| --- | --- | --- | --- | --- | --- | --- | --- |
| 1 | Male | 53 | 37.2 | Left | II | 4.5 | Renal vein |
| 2 | Male | 63 | 25.6 | Right | II | 13.5 | Inferior vena cava |
| 3 | Male | 85 | 28.4 | Right | II-III | 6.2 | Inferior vena cava |
| 4 | Female | 62 | 21.6 | Right | IV | 9.5 | Inferior vena cava |
| 5 | Female | 62 | 28.9 | Right | II | 10.5 | Inferior vena cava |

Supplementary Table 2. Baseline characteristics of ccRCC patients and healthy controls for urinary mass spectrometry

| Parameter | ccRCC patients (n=12) | Healthy controls (n=11) | P value |
| --- | --- | --- | --- |
| Gender, n (%) |  |  | 0.692 |
| Male | 9 (75.0) | 9 (81.8) |  |
| Female | 3 (25.9) | 2 (18.2) |  |
| Age, years, mean ± SD | 59 ± 10.2 | 63.9 ± 6.7 | 0.191 |
| BMI, kg/m², mean ± SD | 24.1 ± 3.1 | 24.2 ± 1.9 | 0.882 |
| Personal history, n (%) |  |  |  |
| Smoking | 3 (25.0) | 4 (36.3) | 0.554 |
| Drinking | 7 (58.3) | 6 (54.5) | 0.855 |
| Diabetes | 1 (8.3) | 1 (9.1) | 0.949 |
| Hypertension | 5 (41.7) | 5 (45.5) | 0.855 |
| Hyperlipidemia | 2 (16.7) | 3 (27.3) | 0.538 |
| Maximum tumor diameter, cm, mean ± SD | 4.1 ± 1.7 | NA |  |
| Tumor laterality, n (%) |  |  |  |
| Left | 4 (33.3) | NA |  |
| Right | 8 (66.7) | NA |  |
| WHO/ISUP grade, n (%) |  |  |  |
| I | 1 (8.3) | NA |  |
| II | 10 (83.4) | NA |  |
| III-IV | 1 (8.3) | NA |  |
| Pathological stage, n (%) |  |  |  |
| T1a | 8 (66.7) | NA |  |
| T1b | 3 (25.0) | NA |  |
| ≥T2 | 1 (8.3) | NA |  |

Supplementary Table 3. Baseline characteristics of ccRCC patients for urinary ELISA analysis

| Parameter | Value |
| --- | --- |
| Gender, n (%) |  |
| Male | 41 (75.9) |
| Female | 13 (24.1) |
| Age, years, mean ± SD | 56 ± 12 |
| BMI, kg/m², mean ± SD | 24.4 ± 3.5 |
| Tumor laterality, n (%) |  |
| Left | 23 (42.6) |
| Right | 31 (57.4) |
| Maximum tumor diameter, cm, mean ± SD | 4.4 ± 3.7 |
| WHO/ISUP grade, n (%) |  |
| I | 2 (3.7) |
| II | 41 (75.9) |
| III | 10 (18.5) |
| IV | 1 (1.9) |
| Pathological stage, n (%) |  |
| T1a | 36 (66.7) |
| T1b | 10 (18.5) |
| T2a | 2 (3.7) |
| T2b | 0 |
| T3 | 5 (9.2) |
| T4 | 1 (1.9) |
| Therapy approach, n (%) |  |
| Radical nephrectomy | 21 (38.9) |
| Nephron-sparing surgery | 31 (57.4) |
| Cryosurgery | 2 (3.7) |
| Time from diagnosis, months, mean ± SD | 8.9 ± 1.9 |
